# Does Late Maturity Alpha-Amylase Impact Wheat Baking Quality?

**DOI:** 10.3389/fpls.2018.01356

**Published:** 2018-09-07

**Authors:** Marcus Newberry, Alexander B. Zwart, Alex Whan, Jos C. Mieog, May Sun, Emmett Leyne, Jenifer Pritchard, Sergio Nicolas Daneri-Castro, Kutty Ibrahim, Dean Diepeveen, Crispin A. Howitt, Jean-Philippe F. Ral

**Affiliations:** ^1^Agriculture and Food, Commonwealth Scientific and Industrial Research Organisation, Canberra, ACT, Australia; ^2^Data61, Commonwealth Scientific and Industrial Research Organisation, Canberra, ACT, Australia; ^3^Department of Primary Industries and Regional Development, South Perth, WA, Australia

**Keywords:** late maturity alpha-amylase, baking, Falling Number, wheat, end product quality

## Abstract

Late maturity α-amylase (LMA) and pre-harvest sprouting (PHS) are both recognized as environmentally induced grain quality defects resulting from abnormally high levels of α-amylase. LMA is a more recently identified quality issue that is now receiving increasing attention worldwide and whose prevalence is now seen as impeding the development of superior quality wheat varieties. LMA is a genetic defect present in specific wheat genotypes and is characterized by elevated levels of the high pI TaAMY1 α-amylase, triggered by environmental stress during wheat grain development. TaAMY1 remains present in the aleurone through the harvest, lowering Falling Number (FN) at receival, causing a down-grading of the grain, often to feed grade, thus reducing the farmers’ income. This downgrading is based on the assumption within the grain industry that, as for PHS, a low FN represents poor quality grain. Consequently any wheat line possessing low FN or high α-amylase levels is automatically considered a poor bread wheat despite there being no published evidence to date, to show that LMA is detrimental to end product quality. To evaluate the validity of this assumption a comprehensive evaluation of baking properties was performed from LMA prone lines using a subset of tall non-Rht lines from a multi-parent advanced generation inter-cross (MAGIC) wheat population grown at three different sites. LMA levels were determined along with quality parameters including end product functionality such as oven spring, bread loaf volume and weight, slice area and brightness, gas cell number and crumb firmness. No consistent or significant phenotypic correlation was found between LMA related FN and any of the quality traits. This manuscript provides for the first time, compelling evidence that LMA has limited impact on bread baking end product functionality.

## Introduction

Alpha-amylases (EC 3.2.1.1) are endohydrolases that cleave α-1,4 glucosidic bonds breaking down starch macromolecules into smaller polysaccharides. Many biological and industrial processes, such as mammalian digestion, plant metabolism, biofuel production, baking, fermentation, and malting rely on the hydrolysis of native starch by α-amylase (Janecek et al., 2014).

Alpha-amylase is considered to be one of the primary enzymes responsible for starch degradation in cereals and particularly wheat. During grain germination, α-amylase initiates the conversion of starch into simple sugars to fuel embryo and coleoptile growth in the first few days of germination. At least four α-amylase isoforms have been described in wheat grain but only a subset of specific isoforms are involved either in grain development or in the germination process (Mieog et al., 2017).

Alpha-amylase has been consistently used by the baking industry to improve dough properties and end product quality (He and Hoseney, 1991). Several studies have demonstrated the beneficial effect of α-amylase on bread texture and elasticity (Patel et al., 2012; Barrera et al., 2016). However, detection of elevated levels of endogenous α-amylase in the grain has been considered as a serious grain quality defect due to its presumed effect on end-product quality (Buchanan and Nicholas, 1980). Premature production of α-amylase during grain development is the result of two different conditions that are considered quality defects by the wheat industry: (1) PHS and (2) LMA. Although PHS is considered an important issue by both wheat breeders and growers globally, it is only in Australia and the United Kingdom over several decades that LMA has been regarded as a significant grain quality defect, although more recently LMA has been considered an issue in other countries (for review Mares and Mrva, 2014).

Pre-harvest sprouting is a genetic condition that breaks grain dormancy when environmental triggers prior to harvest (usually heavy rainfall) cause the grain to germinate while still on the head (Gubler et al., 2005). Sprouted grain is unacceptable as milling wheat leading to it being downgraded to animal feed quality with a resulting decrease in value. Domestication and modern conventional breeding aims to reduce grain dormancy by encouraging rapid and uniform plant establishment, however, this has inadvertently favored PHS susceptibility and occurrence. Thus developing PHS resistance has become a high priority for breeders (Martinez et al., 2018).

Late maturity alpha-amylase is a genetic defect present in specific wheat genotypes and is characterized by abnormally elevated levels of a single α-amylase (TaAMY1) isoform in the aleurone layer during grain development through to harvest (Mares and Mrva, 2008). A recent study has also highlighted the potential involvement of TaAMY4 in the LMA phenotype (Mieog et al., 2017). This accumulation of α-amylase has no detrimental effect on grain composition, morphology, dormancy, or germination. Since the Green Revolution, which enhanced yield and reduced the height of wheat plants through the introduction of the semi-dwarf genes (Rht-D1), LMA has become a stochastically induced genetic defect triggered by environmental conditions (Gooding et al., 2012; Mieog et al., 2017). In Australia, a cold shock at a specific developmental stage induces LMA, while studies in the United Kingdom have described a similar phenotype following a heat shock (Farrell and Kettlewell, 2008).

Elevated levels of α-amylase in the grain reduce the value produced by the FN test. The FN test was developed by Hagberg (1960) as a rapid, high throughput method for determining α-amylase activity in grain and later adopted by industry as a test for sprout-damaged grain (Best and Muller, 1991). In Australia, if low FN is detected, there is a potential $AUS20–50/t penalty to growers due to downgrading superior milling wheat classes to feed grade (Kingwell and Carter, 2017). Therefore the premature production of α-amylase during grain development known as LMA has been considered the major quality problem affecting both growers and breeders in the Australian wheat industry (Lunn et al., 2001; Mares and Mrva, 2014). In the past few years the United States and France have encountered elevated α-amylase levels at harvest without signs of sprouting or starch damage, which suggests that LMA prone lines are present in all breeding programs worldwide. In the United States, the Pacific North West was severely impacted during the 2016 harvest, with losses estimated to be in the order of $US140 million (Bettge, 2018).

Despite the increasing importance and negative economic impacts of LMA to grain growers, to date only one study has been conducted to understand the mechanism underlying expression of LMA (Barrero et al., 2013). Unlike PHS with its widely acknowledged and clearly demonstrated detrimental effects on end-use quality, there is no direct evidence that elevated levels of α-amylase have negative effects on end-use quality (Ral et al., 2016, 2018b).

The current strategies to mitigate, and if possible eliminate LMA revolve around efforts to understand the genetic basis of LMA (Mrva and Mares, 2001; Mohler et al., 2014; Mieog et al., 2017; Milczarski et al., 2017; Borner et al., 2018). These LMA strategies have been put in place despite a clear knowledge gap around the real impact of LMA on wheat flour end product quality. One of the key issues that has impeded developing an understanding of the impact of LMA on end product quality has been the difficulty in inducing LMA under controlled conditions, on a scale large enough to produce sufficient grain for baking studies. The stochastic nature of LMA expression (combination of genetic, environmental conditions and plant developmental stage) makes the prediction of LMA occurence very cumbersome during LMA dedicated field trial (Mares and Mrva, 2014). To overcome this limitation we apply a novel approach focussed on the phenotypic aspects of LMA via exclusively studying the less economically significant constitutive LMA expressing non-Rht tall lines to assess the impact of LMA related elevated α-amylase on baking quality (Gooding et al., 2012).

This study involves a comprehensive assessment of LMA prone lines using a subset of LMA constitutive expressers from a MAGIC wheat population (Huang et al., 2012). LMA expression levels, FN test, total α-amylase activity and a LMA-specific antibody test available in Australia were determined, along with quality parameters including end product functionality such as bread loaf volume, bread slice gas cell number, size, and structure.

This study provides the first direct evaluation as to whether elevated levels of α-amylase caused by LMA impact baking quality.

## Materials and Methods

### Biological Material and Design

Three sample sets were obtained from subsets of tall lines taken from a MAGIC 4-parent wheat population grown at three different site and year combinations: Yanco (34.6° S, 146.4° E) and Narrabri (30.3° S, 149.8° E) during growing season 2009 and 2010 (irrigated, very favorable conditions); and Wongan Hills (30.9° S, 116.7° E, low yielding conditions) during the growing season 2011. In this study and for the baking study, 72 non-Rht LMA prone genotypes were selected from Yanco, 101 genotypes from Narrabri and 67 genotypes from Wongan Hills. For each site, all genotypes were grown on similar size plots according to the local best practice. The LMA test comparison was performed on wholemeal samples from Yanco and included 84 lines for the Stirring Number (SN) test and 196 lines for the ELISA Test, total α-amylase activity assay and TaAMY1 expression level.

All three sites were planted according to partially replicated (*p*-rep) spatially optimized statistical designs and involved a range of 4-parent MAGIC population genotypes including both non-Rht (tall) and Rht (dwarf, semi dwarf) phenotypes. The original intent of these experiments was to utilize the genetic breadth of the MAGIC 4-parent population without any particular focus upon the influence of Rht lines on the quality traits. Consequently the tall non-Rht lines were randomly distributed throughout these experimental designs, including the milling, baking, and flour quality assessment designs. However, although the role of tall non-Rht lines on wheat and flour quality were not a specific objective of the original experimental designs, the ability to utilize quality data from these tall lines to aid in understanding the impact of LMA upon end-product quality was deemed sufficiently important to undertake an investigation using subsets of the 4-parent lines from the complete experimental designs.

Grain samples from Yanco 2009 were milled to produce wholemeal and white flour samples. Grain samples from Narrabri 2010 and Wongan Hills 2011 were milled to produce white flour samples. To reduce the time and cost of milling white flour samples from the three large field trials, a proportion of the white flour samples were milled from composites of grain from two plots growing the same genotypes (Ral et al., 2018a). Hence, a proportion of the white flour samples represent a blending of the environmental effects in the field.

### Milling and Flour Preparation

White flour from grain (2 kg) samples from the three sites were milled at commercial testing laboratories using laboratory scale Bühler MLU-202 pneumatic laboratory mills (Bühler AG, Uzwil, Switzerland). Grain samples were conditioned to 14% moisture content, white flour was derived from blending the B1, B2, B3, R1, R2, and R3 mill fractions. The optimal water absorption levels of the flours were determined on small-scale 4 g micro-doughLAB z-arm mixers (Perten Instruments, Sydney, NSW, Australia).

For the Yanco site grain (4 g) was milled into wholemeal flour using an UDY Cyclone Sample Mill (UDY Corporation, Fort Collins, Co., United States) fitted with a 0.5 mm screen without any prior moisture conditioning.

### Alpha-Amylase Assay

Alpha-amylase activity was determined in 10 mg samples using the CERALPHA kit (Megazyme International Ireland Ltd.), with the manufacturer’s protocol adapted for 96-well format and with appropriate dilutions (Whan et al., 2014). Alpha-amylase activity is expressed in Ceralpha-unit per gram of flour as per the manufacturer’s recommended methods.

### ELISA Assay

Late maturity alpha-amylase testing was performed on protein extracted from 10 mg wholemeal flour according to the method described by (Verity et al., 1999; Barrero et al., 2013). All spectrophotometric measurements were performed using a Thermo Scientific Multiskan Spectrum plate reader.

### RT-qPCR TaAMY1 Expression Level

RNA was extracted using the protocol described in Mieog et al. (2017). Amylase-isoform specific primers for real-time quantitative PCR (RT-qPCR) were developed for TaAMY1 as previously described for qPCR (Mieog et al., 2013). RT-qPCR runs were performed on the MyIQ real-time PCR system (Bio-Rad) using SensiFAST one-step RT-qPCR kits containing a mastermix, reverse transcriptase and RNAse inhibitor (Bioline). A typical reaction consisted of 10 μL mastermix, 4.6 μL primer premix (1.6 μM each), 0.2 μL reverse transcriptase and 0.4 μL RNAse inhibitor and 5 μL RNA sample. Primers, plasmids of TaAMY1 and TaActin, obtained during sequencing or from cloned RT-qPCR products, were mixed in equal copy numbers and used as a calibrator sample to be able to directly compare TaAMY1 expression levels according to Mieog et al. (2017).

### Falling Number Determination

The eFN of the tall lines was determined using the RVA SN test (AACC International Approved Methods 22-08.01, 1995) on 4 g of wholemeal flour (for Yanco wholemeal samples) and 3.5 g of white flour for each of the three sites using a Rapid Visco Analyser RVA-4SA (Perten Instruments, Sydney, NSW, Australia). The eFN was estimated from the measured SN values as per the formula of Barnard et al. (2005).

### Baking

The flour samples were subjected to a long fermentation straight dough bread-making process that is used internationally for determining the baking quality of wheats. The method was based upon AACC Approved Methods 10-09.01 and 10-10.03 (AACC International Approved Methods 10-09.01, 2008; AACC International Approved Methods 10-10.03, 2009). A doughLAB mixer with 300 g bowl (Perten Instruments, Sydney, NSW, Australia) was used to mix the doughs to peak dough consistency. The ingredients were added to 250 g of flour at the following percentages of flour weight: dried yeast 0.7% (Mauripan), vegetable fat 2% (Doveg Shortening), sugar 1%, salt 1%, baking improver 0.5% (Straight Dough Improver), and water at the previously determined optimal water absorption level of less than 4%. The sugar and salt were domestic grade, while the dried yeast, vegetable fat and baking improver were all commercial baking ingredients with the latter two sourced from George Weston Foods (Sydney, NSW, Australia) and the yeast obtained from Mauri ANZ (Sydney, NSW, Australia). The doughs were mixed at 180 rpm to optimal dough development as determined by the baker, with mixing times ranging from 2.03 to 7.00 min. The doughLAB bowl temperature was maintained at 18.5–19°C to attain a final dough temperature of 26–28°C. Following mixing the dough was scaled into two 150 g pieces and placed into sealed containers and held at 28°C in a fermentation cabinet for 105 min. After passing the dough pieces through a bread molder (Mono Mini Moulder, MONO Equipment, Swansea, Queensway, United Kingdom) the dough pieces were returned to the sealed plastic container and fermented for a further 50 min at 28°C, after which they were again run through the bread molder, returned to the sealed container and subjected to another 28°C fermentation for a further 25 min. The dough pieces were then passed through the bread molder a final time before placing into baking tins and given a final proof at 35°C and 85% humidity for 50 min. The proofed doughs were baked at 230°C for 20 min. The baked loaves were cooled at room temperature for an hour then loaf volumes were determined using rapeseed displacement (AACC International Approved Methods 10-05.01, 2000), the weight of each loaf was also measured. Oven spring was determined from the difference in height of the baked loaf while still in the baking tin less the height of the proofed tinned dough measured immediately prior to placing into the oven. The crumb firmness and structure were measured 1 day after baking. To prevent drying out the loaves were stored in sealed plastic bags at room temperature immediately after loaf volume was measured. Immediately before measuring the firmness and structure of the loaves the breads were sliced into 14 mm thick slices. The crumb structure was determined on the middle two slices using image analysis (C-Cell Bread Image Analyser, Calibre Control International, Warrington, United Kingdom) immediately followed by crumb texture analysis. The Slice Area, Slice Brightness, and Number of Cells (number of gas bubbles per slice) measurement parameters for the two slices per loaf were extracted from the C-Cell data. Bread firmness was determined on the same two slices using TA.XTplus Texture Analyser instruments (Stable Micro Systems, Godalming, United Kingdom) utilizing a 0.5 inch diameter Delrin probe and the standard Texture Profile Analysis (TPA 1 method) double compression cycle was used (pre-test speed 1 mm⋅s^-1^, test and post-test speeds 5 mm⋅s^-1^, 10 mm compression distance, 5 g trigger force). The Crumb Firmness was determined from the maximum height of the second compression peak.

### Statistical Analysis

The data obtained were collated into two datasets. The white flour dataset contained data for eFN and the bread quality parameters oven spring, loaf volume and weight, number of cells, slice area, crumb firmness and slice brightness. The wholemeal flour dataset contained data for eFN, LMA-ELISA test, and TaAMY1 expression level parameters. LMA-ELISA test, total α-amylase activity and TaAMY1 parameters were transformed by applying a base-2 logarithm prior to analysis.

For each parameter, there were field, milling and testing stages to the data collection process, with an associated statistical randomisation of the lines and grain/flour samples to the respective field/milling/testing layout. Bivariate linear mixed model (LMM) analyses were used to partition the effects at the milled grain sample level from other sources of variation produced by baking and testing stages, and hence estimate correlations between milled grain sample effects for relevant pairs of parameters. The LMM analyses also allowed us to account for possible extraneous effects in the analysis (such as “day of processing of sample” effects) in the milling and testing phases, effectively removing their influence from the estimation of effects and effect correlations. Models were fitted using the ASReml-R LMMs software (Butler et al., 2009). Milled samples were fitted as random effects, leading to BLUPs as estimates of the milled sample-level effects for each response, along with the estimated correlation and an associated *p*-value derived using the REML likelihood ratio test for assessing the statistical significance of the correlation estimate (Butler et al., 2009).

The interest here is in the correlations occurring at the level of the milled flour samples, hence the models used to estimate milled sample-level correlations in the white flour dataset did not model genotypic effects or account for environmental effects in the field trial, since these effects simply contribute to the variation observed between milled flour sample effects. An exception to the above analysis procedure was the estimation of correlations between eFN and LMA-ELISA, total α-amylase activity or TaAMY1 expression parameters in the wholemeal dataset. No replication of samples was performed when determining eFN in the wholemeal samples, hence only one eFN observation was obtained per milled sample. Consequently, milled sample-level effects cannot be completely isolated from other sources of variation introduced in the eFN testing process. Hence, reported correlations between eFN and LMA-ELISA, total α-amylase activity or TaAMY1 expression parameters are simple Pearson correlations between the eFN observations and milled sample level BLUPs, and the *p*-value is obtained from the standard *t*-test for a Pearson correlation rather than the REML likelihood test.

## Results

### LMA Trait Measurements and Correlations

Subsets of tall non-Rht lines taken from a MAGIC 4-parent wheat population from the Yanco trial were selected to perform a complete LMA assessment including SN converted eFN, total α-amylase activity assay, ELISA based high pI α-amylase detection kit and RT-qPCR measurement of expression of the transcript expressed in LMA affected wheat, TaAMY1.

**FIGURE 1:**
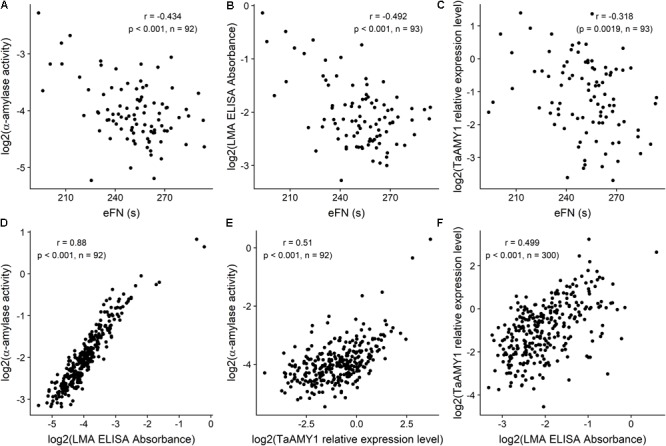
Scatterplots and associated correlations (*r*) between equivalent Falling Number (eFN), and total a-amylase activity **(A)**, LMA ELISA Absorbance **(B)**, and TaAMY1 relative expression level **(C)** milled sample-level effects; between total a-amylase activity, and LMA ELISA Absorbance **(D)** and TaAMY1 relative expression level **(E)**; between TaAMY1 relative expression level and LMA ELISA Absorbance **(F)** for wholemeal flour samples from the Yanco site. Falling Number is expressed in seconds. LMA Elisa is expressed as absorbance according to Verity et al. (1999) and TaAMY1 is expressed as relative expression to Actin (Log2). Amylase assay responses were available for *n* = 300 milled samples, but only *n* = 93 samples were available for eFN assessment. Plotted amylase assay responses are milled sample-level Best Linear Unbiased Predictors (BLUPs) from linear mixed model analyses of the data, while eFN values are based on raw stirring number data only.

For this comparative LMA assessment population, eFN ranged from 193 to 294 s. Total α-amylase activity ranged from 0.002 to 1.977 Ceralpha unit per gram of flour. LMA values in the wholemeal flour ranged from 0.04 to 3.09 according to the ELISA test; and TaAMY1 relative expression to actin ranged from 0.03 to 11 (**Figure 1**; Ral et al., 2018a).

Among these samples, eFN was significantly negatively correlated with both total α-amylase activity (-0.434, *p* < 0.001), ELISA Test (-0.492, *p* < 0.001) and RT-qPCR (-0.318, *p* = 0.00187). Total α-amylase activity correlated highly with ELISA test (0.88, *p* < 0.001) and significantly with RT-qPCR (0.51, *p* < 0.001). ELISA and RT-qPCR were significantly positively correlated (0.499, *p* < 0.001) (**Figures 1A–F**).

These significant correlations between the four existing LMA tests confirmed the LMA nature of the tall population. For practical reasons and to reproduce the standard testing method for LMA the decision was made to focus on the most current and high throughput test, the effective FN as a surrogate for LMA identification in the white flour samples.

### Baking Trait Measurements and Correlations

In this study 72 non-Rht LMA prone genotypes were selected from Yanco, 100 genotypes from Narrabri and 67 genotypes from Wongan Hills.

White flour baking performance including oven spring, bread loaf volume and weight, cell number, slice area and brightness and crumb firmness were assessed using an internationally approved AACCI methods.

The range of baking performance across the non-Rht population was compared to those from the MAGIC semi-dwarf genotypes and the set of elite cultivars included in the three site MAGIC trials (**Supplementary Figure S1**). The data show no statistical difference across the seven traits indicating that the baking performance of the tall lines did not differ from the semi-dwarf nor the commercial elite lines grown at the three sites.

**FIGURE 2:**
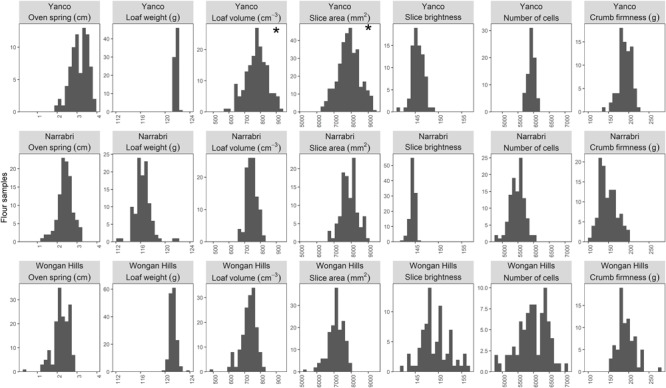
Milled sample-level BLUP distributions for the baking quality traits for the Yanco, Narrabri, and Wongan Hills sites are presented as histogram. Subplots marked with an asterisk display raw trait data as the LMM analysis was unable to estimate any non-negligible variation between milled samples.

The milled sample-level BLUP distributions of the baking traits for the three sites are presented as histograms in **Figure 2** and in the data repository (Ral et al., 2018a).

The baking traits were broadly similar across the three sites. Oven spring values, the increase in loaf volume during the baking stage, were similar for Narrabri and Wongan Hills, whereas the Yanco site exhibited a slightly larger oven spring. The loaf weights from the Yanco and Wongan Hills sites were similar, while those of the Narrabri site were slightly lighter and more variable. Loaf volumes could not be estimated for the Yanco site using the prediction model (see section “Materials and Methods”) however, both the Narrabri and Wongan Hills sites displayed very similar loaf volumes. As with loaf volume the slice area of the bread could not be estimated for the Yanco site using prediction model, while the Narrabri and Wongan Hills sites had similar slice areas, although the Narrabri sites range extended to slightly larger slice areas. The slice brightness profiles were similar for the Narrabri and Yanco sites, while, the slice brightness measurements were more variable for Wongan Hills with the presence of much brighter slice values. The number of cells is a measure of the number of gas cells per bread slice and for the Straight Dough breadmaking method employed here greater numbers of small gas cells are preferable. Again the number of cells for the three sites was broadly similar, however, the Wongan Hills samples exhibited a much greater degree of variation with bread samples possessing gas cell numbers that were much greater than those observed at either the Yanco or Narrabri sites. The crumb firmness profiles were broadly similar across all three sites, although the Narrabri site did have some samples that were slightly less firm than the other two sites.

Among flour samples from tall lines at all three sites, eFN milled sample-level BLUPs ranged from ∼157 to ∼234 s with 250–300 s being the international average standard for FN of an acceptable grain sample.

**FIGURE 3:**
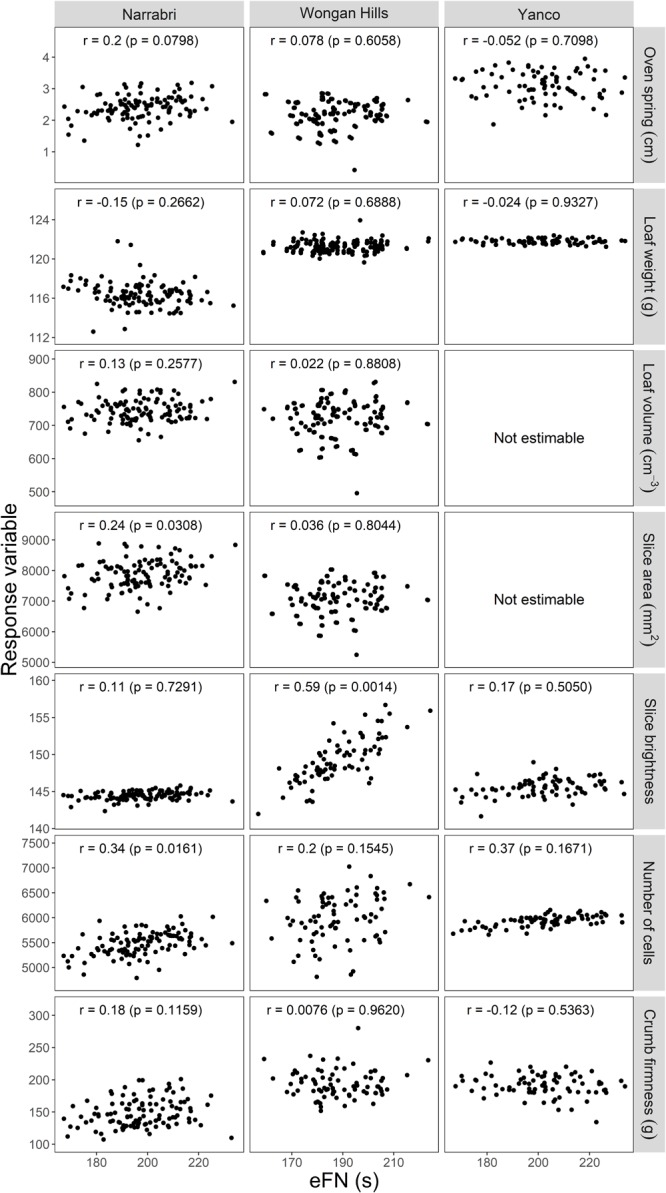
Scatterplots of milled-sample level BLUPs and associated correlations (*r*) between equivalent Falling Number (eFN) and bread quality traits for the three sites, Yanco, Narrabri, and Wongan Hills, with associated REML likelihood ratio test *p*-values. Associated numbers of plotted observations are *n* = 77 (Yanco), *n* = 108 (Narrabri), and *n* = 78 (Wongan Hills). BLUPs for loaf volume and slice area at Yanco could not be estimated (see Section “Results”).

Milled sample correlations between eFN and baking traits were not significantly different from zero at the *p* < 0.05 threshold, except for slice brightness at Wongan Hills (0.59, *p* = 0.0014), number of cells at Narrabri (0.34, *p* = 0.0161) and slice area at Narrabri (0.24, *p* = 0.0308), as summarized in **Figure 3** and data repository (Ral et al., 2018a). However, those correlations were not consistent across the three sites for those specific traits.

Quality responses in **Figure 3** exhibit small, though non-negligible, variation at the milled sample level, indicating that the variation in the phenotype was dominated by that introduced in the baking and quality testing steps.

For the parameters loaf volume and slice area in the Yanco 2009 trial, variation due to differences between milled samples (the milled sample-level effects) was negligible compared to variation introduced by the baking and quality testing steps of data collection process. Consequently, the LMM analysis could not estimate non-negligible variation between the milled samples, nor estimate the corresponding correlations with eFN. This is why, in **Figure 2**, the corresponding panels have been replaced by standard phenotypic correlation and no scatterplots are been presented in **Figure 3** for these two traits.

With the exception of the above mentioned traits, the correlations between baking traits and eFN were on average not significant across the three sites.

## Discussion

The original intent of this study was to utilize the genetic breadth of the MAGIC 4-parent population without any particular focus upon the influence of Rht lines on the quality traits.

Although the role of tall lines on wheat and flour quality was not a specific objective of these experimental designs, the ability to utilize quality data from these lines to aid in understanding the impact of LMA upon end-product quality was deemed sufficiently important to extract the data for this investigation.

With the selection of tall lines and in the absence of any evidence of sprouting, it was reasonable to assume that the reduced eFN was due to LMA rather than sprouting damage. However, we decided to confirm the LMA basis of the low eFN with LMA assessment methods (Mohler et al., 2014; Borner et al., 2018). Each reference test (including total α-amylase assay, ELISA test, RT-qPCR and SN), was performed on wholemeal flour to avoid any reduction of TaAMY1 protein or *TaAMY1* mRNA due to pearling (Chakraborty et al., 2003). In addition, Kweon et al. (2010) demonstrated the strong correlation between viscosity tests performed on wholemeal and related white flour samples.

For each of the alternative LMA assessment methods, clear correlations with eFN, demonstrating the involvement of TaAMY1 and LMA in the selected MAGIC lines were found. The correlation between the total α-amylase activity and the ELISA test, detecting TaAMY1 was strong confirming the sole involvement of TaAMY1 as active determining enzyme in the LMA phenotype. Mieog et al. (2017) has suggested the potential involvement of a newly discovered TaAMY4 in the LMA phenotype. This hypothesis was based on the significant co-expression of *TaAMY1* and *TaAMY4* in dry seed from LMA prone lines. While the mechanism through which TaAMY4 could be involved in the LMA phenotype remains to be elucidated, the result suggests that most of the total α-amylase activity in LMA affected dry grain is generated by TaAMY1. However, a role of an active TaAMY4 prior to grain maturation or during germination cannot be excluded.

The correlations involving *TaAMY1* expression level were the least convincing. Several factors could explain these weaker correlations. Determination of relative expression level required extraction of fragile total RNA that could introduce variation. In addition, Barrero et al. (2013) suggested a narrow window for TaAMY1 expression when LMA is triggered in an Rht background. Mieog et al. (2017) found elevated presence of TaAMY1 mRNA in LMA lines. The significant correlation found between RT-PCR and eFN clearly indicated that mRNA survives in dry grain but could be partially degraded during grain ripening.

The viscosity measurements performed on white flour (SN) gave a wide range of viscosities, corresponding to an eFN range of 157–234 s and 193–294 for the Yanco wholemeal flours. The result of lower eFN values for white flour compared to the Yanco wholemeal subsamples were unexpected. The milling process separates the bran from the flour and reduces the amount of aleurone specific enzymes thus increasing FN (Chakraborty et al., 2003). Therefore a higher FN is usually expected for white flour compared to wholemeal. However, several factors could explain this difference including a difference in the storage condition between white flour and grain. Brandolini et al. (2010) showed that storage temperature could impact the level of α-amylase activity. The difference in storage temperature between white flour and wholemeal in our experiment could explain the variation. The nature of the test sample could have also induced some variation. Viscosity test on white flour was based on 2 kg aliquots while wholemeal data was generated on samples of several grams. Because eFN and baking study were performed for the same low eFN white flour, this difference has no consequence on the study.

According to Mares and Mrva (2008), samples affected by LMA displayed a FN somewhere between 150 s and the acceptable threshold [from 250 to 350 s depending on the wheat grade and growing region, and country wheat quality receival standard according to Posner and Hibbs (2011)].

This study attempted to correlate eFN with seven baking quality attributes. However, no clear correlation could be found between any of the baking properties with the exception of slice brightness only for the Wongan Hills site (0.59). This strong correlation could illustrate what has been described as Maillard Reaction (Sanz-Penella et al., 2014; Ral et al., 2016; Marti et al., 2017). This reaction is directly related to the impact of α-amylase on starch conversion. Increased levels of α-amylase in flour accelerates soluble sugar release. These soluble sugars polymerise with amino acids toward the end of the baking process causing crumb browning. Another plausible explanation is that elevated α-amylase levels generated greater quantities of fermentable sugar leading to high CO_2_ retention and altering gas cell formation due to enhanced yeast fermentation (Marti et al., 2017).

While association between eFN and slice brightness was expected, the lack of any strong and significant correlations was noteworthy. This result strongly suggested some limitation of the FN test in LMA detection. This limitation was also suggested by the correlation obtained with the wholemeal flour between FN and the other LMA tests available. Although the correlations between eFN and the other tests were highly significant, the correlations were not strong or at least not as strong as expected for a test that was created to detect α-amylase. While the FN test assesses viscosity of the flour slurry, it does not directly measure α-amylase activity. FN measures are affected by changes in the physical properties in the starch portion of the wheat grain during the test. However, in addition to α-amylase activity, several other factors can impact flour gelling properties, and therefore FN, including environment (Barnard and Smith, 2012) and starch structure. Starch composition, amylose-amylopectin ratio and chain structure can greatly affect the starch viscosity. Luo et al. (2015) have shown wide variations in starch viscosity among rice mutants affected by several starch metabolism enzymes. In potato, RNA interference of Glucan Water Dikinase caused reduction in tuber starch phosphate content associated with a clear reduction in viscosity (Viksø-Nielsen et al., 2001).

According to Lindahl and Eliasson (1992), protease and endopeptidase can also degrade the gluten matrix thus reducing wheat flour gelling properties. Gluten integrity is thus also affected during sprouting but as no proteases are expressed with LMA, gluten integrity remains unaffected (Simsek et al., 2014). The FN test is used to detect sprout damage where both α-amylase and protease are simultaneously expressed, but the presence of either α-amylases or proteases is sufficient to warrant a low FN especially in case of sprout damage (Edwards et al., 1989). The more significant role played by proteases over elevated α-amylases in decreasing end-product quality has been demonstrated a study comparing flours milled from germinated grain and grain possessing transgenically elevated levels of α-amylases. This study on noodle quality revealed that germination does impact gluten integrity in wheat grain thus downgrading noodle quality; however, elevated levels of α-amylase alone did not show any detrimental effect (Ral et al., 2018b). This noodle research followed on from a previous study that showed even greatly elevated levels of a single α-amylase isoform had no detrimental effect on small scale baking quality (Ral et al., 2016).

To summarize, all flours with elevated levels of α-amylase will show a low FN as demonstrated in the wholemeal study with a significant negative correlation between LMA and eFN. However, not every low FN value is related to LMA or high α-amylase level. As LMA is characterized by the sole expression of single type of α-amylase (TaAMY1), this assumption that a decrease in flour paste viscosity measured by the FN test is associated with all the detrimental arsenal of degradative enzymes that typifies sprouting becomes problematic (Lunn et al., 2001).

Several alternative options to definitively identify LMA affected grains have been investigated over the years. Created with the aim of LMA detection, both the ELISA (Verity et al., 1999) and RT-qPCR relative α-amylase gene expression assays specifically target the main enzyme involved in LMA, TaAMY1 (Cheng et al., 2014). These approaches can be useful for breeding programs or wheat variety classification in quantifying the presence of TaAMY1 during grain development (Mares and Mrva, 2008), although the difficulties of triggering LMA in semi-dwarf breeding germplasm remains problematic. However, the methods are too slow and inappropriate for LMA detection at grain receival. During germination and sprouting, many α-amylases are expressed including TaAMY1 (Ainsworth et al., 1985) making the differentiation between LMA and sprouting cumbersome. Even in this study and despite the selection of constitutive LMA expressers, we cannot rule out the potential presence of some sprouted flour in the population.

The lack of correlation between the eFN LMA test and baking traits is particularly noteworthy. The overall total α-amylase activity present in flour samples ranged from near zero for the lowest to 2 Ceralpha unit per gram of flour for the highest. Although being significantly elevated and in accordance with an earlier LMA study (Mrva and Mares, 1999), this overall α-amylase activity remained very low compared to severely sprouted grains (Simsek et al., 2014). In germinated grain total α-amylase level can reach over 50 Ceralpha unit per gram of flour after 2 days (Ral et al., 2018b).

According to previous observations, a two to threefold increase of activity is sufficient to reduce the FN scores below the acceptable limit (300 s in Australia) (Mares and Mrva, 2008). Nevertheless there was no impact on baking. Transgenic wheat lines overexpressing wheat TaAMY3 specifically in the grain, have been shown not to have detrimental effects on either small scale baking quality or white salted noodle firmness despite having a FN close to 60 s (Ral et al., 2016, 2018b). These lines included some with a 30-fold increase in total α-amylase activity. An even greater increase in α-amylase activity results from the addition of commercial baking improver or malt (Marti et al., 2017). Recent studies showed that all four types of wheat α-amylase differ slightly in their protein structure including the presence of additional sugar binding domains suggesting different enzymatic properties (Cockburn et al., 2015; Mieog et al., 2017). While it is reasonable to assume that different types of α-amylase have different enzymatic properties, those differences are likely insignificant compared to the 1000-fold increase in α-amylase associated with the addition of baking improver.

If an elevated level of α-amylase due to LMA has the expected negative impact on baking quality then this would be evident from this large scale baking study involving LMA expressing lines.

Many factors can impact baking properties including genetic, phenotypic, or environmental (Cavanagh et al., 2010). However, if a strong correlation exists between two attributes, this correlation should be detected within the boundaries of the sampled population for 166 tall genotypes and three field sites. In this study there was a clear absence of correlation between LMA related eFN and any of the baking quality traits analyzed. Interestingly a recent study also highlighted the weak correlation between low FN in soft wheat and Japanese sponge cake volume (Kiszonas et al., 2018).

This pilot study further questions whether LMA is detrimental for baking quality. Because of the economic significance of this issue, further investigations are warranted with larger scale wheat flour based product quality testing using multisite dedicated trials including LMA constitutive, sensitive, and resistant varieties. With FN being one of the critical parameters upon which grain quality is assessed for trading wheat, it is understandable that the industry wishes to mitigate and eliminate LMA.

In the absence of any data establishing a detrimental effect of LMA on quality, there are now strong incentives to develop alternative simple, robust and high throughput tests that can discriminate between LMA and PHS. Such a test would need to be quick, inexpensive and appropriate for use at grain receival. One consequence would be to allow the resources of breeders to be used more efficiently in delivering enhanced varieties for growers. Given the importance that LMA has for the global wheat industry and the economic activities and livelihoods that stem from it, a more thorough understanding of the effect of LMA on wheat quality is needed. The findings of this study, together with previous studies, demonstrates that the detrimental impacts of LMA on flour and product quality are difficult to discern and points to the need for further evaluations of this contention and other assumptions surrounding LMA.

## Data Availability

Complete dataset is available in a data access portal (Ral et al., 2018a).

## Author Contributions

MN and J-PR were involved in the design and coordination of the study, conceived and performed the experiments, analyzed the data, and wrote the manuscript. AZ and AW analyzed the data and wrote the manuscript. JM, JP, and SD-C performed the experiments and analyzed the data related to the LMA assessment. MS, EL, and KI performed the experiments and analyzed the data related to the baking quality assessment. DD generated biological material. All authors read and approved the manuscript.

## Conflict of Interest Statement

The authors declare that the research was conducted in the absence of any commercial or financial relationships that could be construed as a potential conflict of interest.

## Supplementary Material

FIGURE S1 Boxplots of raw values for bread quality traits from commercial varieties, as well as tall and semi-dwarf MAGIC RILs grown at Yanco, Narrabri, and Wongan Hills. Horizontal lines indicate the median and interquartile ranges of the observed values.

## Abbreviations

BLUP: best linear unbiased predictions
DW: dry weight
eFN: equivalent Falling Number
FN: Falling Number
LMA: late maturity alpha-amylase
MAGIC: multi-parent advanced generation inter-cross
PHS: pre-harvest sprouting
WT: wild-type
