# Supplementary figures and images for: Does Late Maturity Alpha-Amylase Impact Wheat Baking Quality?
Source: Front Plant Sci. 2018 Sep 7;9:1356. doi: 10.3389/fpls.2018.01356 (PMC6137811; doi:10.3389/fpls.2018.01356)

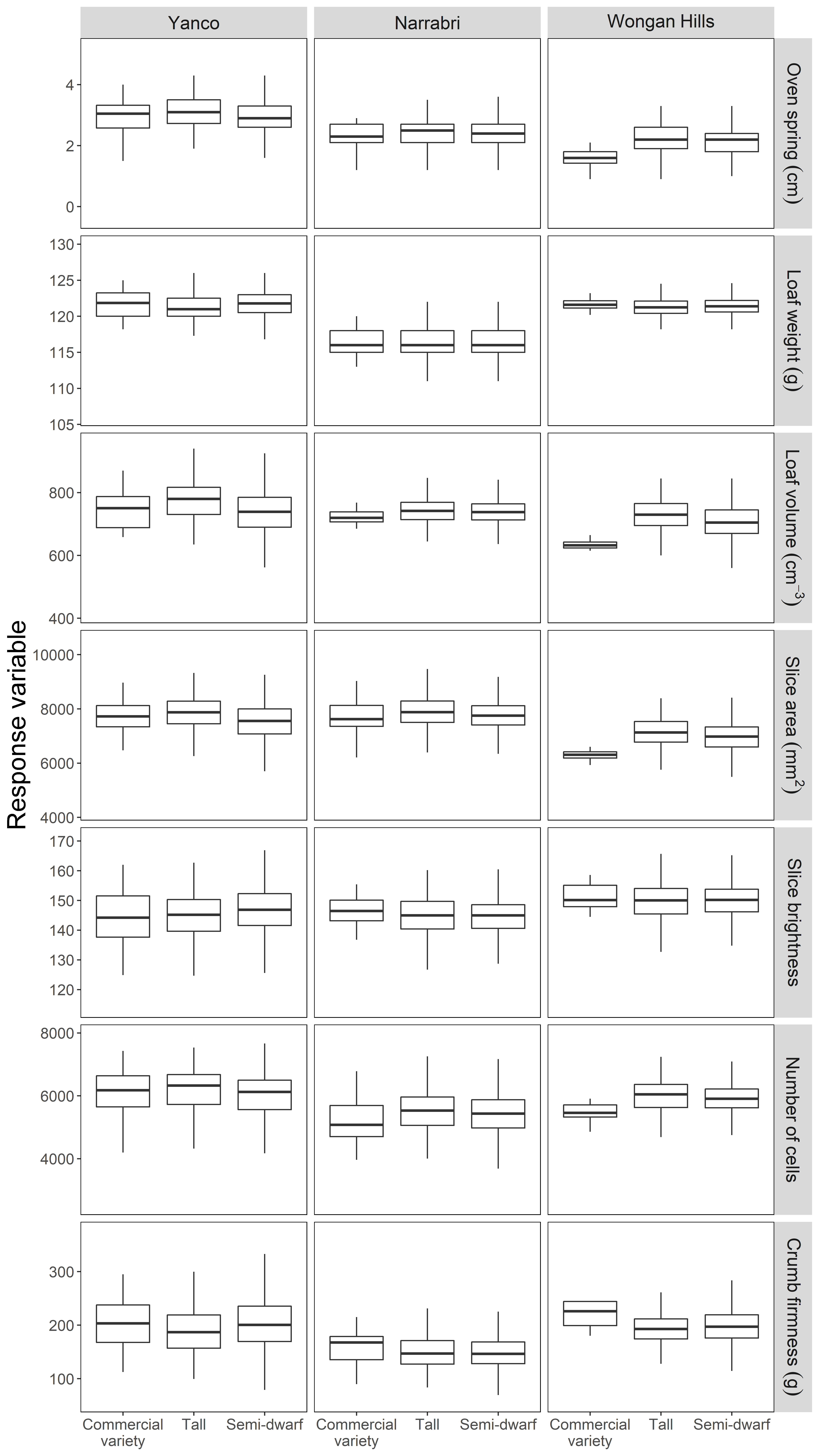

Supplement: FIGURE S1 — Boxplots of raw values for bread quality traits from commercial varieties, as well as tall and semi-dwarf MAGIC RILs grown at Yanco, Narrabri, and Wongan Hills. Horizontal lines indicate the median and interquartile ranges of the observed values. [file Image_1.TIFF]
